# The Plastid Genome of *Deschampsia cespitosa* (Poaceae)

**DOI:** 10.3390/molecules24020216

**Published:** 2019-01-09

**Authors:** Jorge O. Chiapella, Michael H. J. Barfuss, Zhi-Qing Xue, Josef Greimler

**Affiliations:** 1 Instituto Multidisciplinario de Biología Vegetal (IMBIV-CONICET), Universidad Nacional de Córdoba, POB 495, Córdoba 5000, Argentina; 2 Department of Botany and Biodiversity Research, Faculty of Life Sciences, University of Vienna, Rennweg 14, 1030 Vienna, Austria; michael.h.j.barfuss@univie.ac.at (M.H.J.B.); a11844475@unet.univie.ac.at (Z.-Q.X.); josef.greimler@univie.ac.at (J.G.)

**Keywords:** *Deschampsia cespitosa*, *Deschampsia antarctica*, plastid, chloroplast genome comparison, high-throughput sequencing

## Abstract

Plastid genome analysis of non-model organisms provides valuable information for basic research e.g., molecular evolutionary genomics, phylogeny and phylogeography. *Deschampsia cespitosa* is the most widespread species of the genus and it is a common grass that is found across Eurasia and North America. Scattered populations in regions of appropriate ecological conditions are also found in Australia, New Zealand and southern South America, where it is sympatric with *D. antarctica*. We analyzed the plastid genome of a sample of *Deschampsia cespitosa* of the Austrian Alps using high-throughput sequencing. The plastid (cp) genome shows the typical quadripartite structure with a length of 135,340 bp, comprising a large single-copy (LSC) region of 79,992 bp, a small single-copy (SSC) region of 12,572 bp and two inverted repeats (IR) regions of 21,388 bp each. It contains 115 genes, including 85 protein-coding genes, four ribosomal RNA genes and 30 transfer RNA genes. The GC content (%), number of repeats and microsatellites, RNA editing sites and codon usage were highly similar to those of *D. antarctica*. The results of this present study highlight the extremely conserved nature of the cp genome in this group, since the comparison involved individuals separated by about 13,000 km, from the Alps to Antarctica.

## 1. Introduction

The rapid decrease in costs of next-generation sequencing methods has resulted in an increase in the availability of completed plastid (cp) genomes [1], enabling comparisons at the genomic level even between closely related species [2,3]. The amount of cp genomes increased from 800 in 2016 [4] to about 2400 in 2018 [5], which allowing for close examination of features [6] and whole plastid-based phylogenetic relationships [2,7].

Plastid genomes of angiosperms range in size from 120 to 170 kb [8]. The cp genomes are highly conserved in a quadripartite organization, namely, a main large single-copy (LSC), a small single-copy (SSC) region and two inverted repeats (IRs) [9]; functional categories include (i) protein-coding genes, (ii) tRNA coding genes, (iii) introns and (iv) intergenic spacers. The number of genes varies between approximately 100 and 120 [10].

In the grass family (Poaceae), the cp genome size roughly varies from 134 kb in *Oryza sativa* [11] to 140 kb in *Sorghum bicolor* [12]. Until now, the Poaceae have been shown as divided in two major clades: one with three subfamilies (Bambusoideae, Ehrhartoideae and Pooideae; the BEP clade) and the other with seven subfamilies (Panicoideae, Arundinoideae, Chloridoideae, Centothecoideae, Micrairoideae, Aristidoideae and Danthonioideae; the PACCMAD clade) [7]. The BEP clade comprises the majority of the grasses of cold-temperate regions; plastid genome size ranges from 134,5 (*Triticum aestivum*) to 136,5 kb (*Agrostis stolonifera*) [12], with the Antarctic hair-grass (*Deschampsia antarctica*) having an intermediate plastid genome with a size of 135,362 bp [6]. As one of the only two flowering plants (and the only grass) native to Antarctica, *D. antarctica* has been much studied, and studies have focused on the mechanisms of adaptation of the species to the extreme environments of the Antarctic continent. These mechanisms include physiological and morphological attributes [13,14]. Study of genetical aspects has revealed that there is low genetic variation [15] and that Antarctic populations originated from populations in Patagonia [16]. Other studies on *D. antarctica* have included the search of genes expressed under cold stress [17], the later development of cold resistant transgenic rice plants [18] and the resistance to oxidative stress after exposure to UVB radiation [19]. Recently, the effect of Antartina^TM^, which was developed from extracts of *D. antarctica*, has been evaluated as an inhibitor of colorectal carcinoma growth and liver metastasis [20].

A previous phylogenetic analysis [21] showed that *Deschampsia antarctica* to be closely related to *D. cespitosa*; these species are sympatric in Patagonia [22,23] and share many loci with repetitive DNA elements (L. González, unpublished data). Despite the many studies on *D. antarctica*, its relationships at the genomic level are not well understood. Available evidence points to *D. cespitosa* as its closest relative, and, therefore, we focus here on the common species *D. cespitosa* (tufted hair grass), a tussock-forming, wind-pollinated, self-incompatible species with wide morphological variation throughout its large, nearly cosmopolitan distribution. It is mainly distributed in temperate and cold-temperate zones of the Northern Hemisphere, but isolated populations are found in southern South America, Australasia and South Africa [22]. The aims of this study are to (1) present the complete cp genome of *D. cespitosa*, (2) to compare it with *D. antarctica*, and (3) to assess regions of sequence diversity to detect potential markers for genetic, phylogenetic and phylogeographic studies. The understanding of relationships could clarify the evolutionary processes acting between sympatric populations of the two putative species, that have led to one of them undergoing: (1) adaptation to extreme environments; (2) development of morphological differences; and (3) speciation. A comparative analysis of variation in features is presented as a step towards identifying similarities between these taxa. To provide an evolutionary framework, we selected some early-diverging Poaceae and some closely related BEP taxa to include in a phylogenetic reconstruction.

## 2. Results

### 2.1. Sequencing

Illumina paired-end sequencing yielded 11,058,892 raw reads with an average length of 350–400 bp (total 4,423,556,800 bp).

### 2.2. Genome Assembly and Characteristics

**Figure 1:**
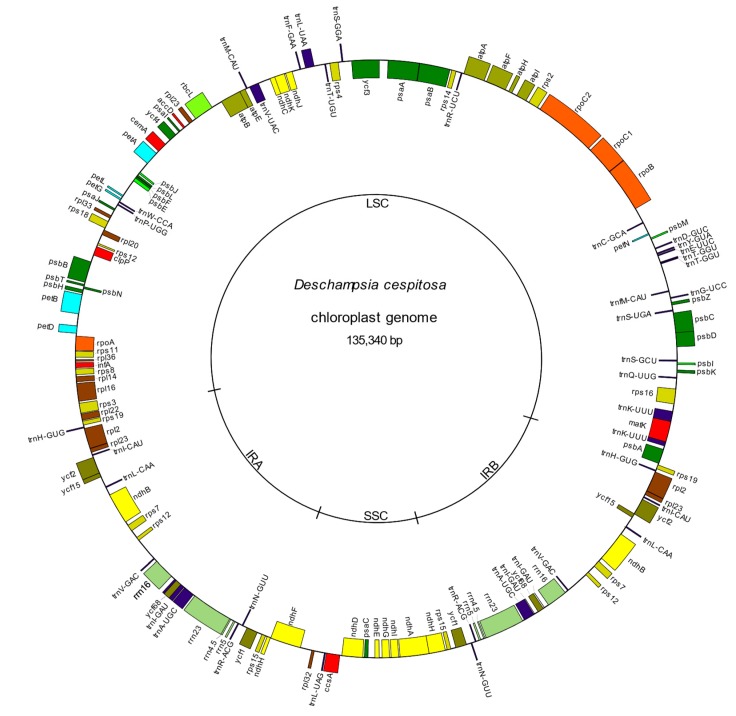
The plastid genome of *Deschampsia cespitosa*. Genes outside and inside the circle are transcribed counterclockwise and clockwise, respectively.

The cp genome of *Deschampsia cespitosa* resulted in a typical circular quadripartite structure of 135,340 bp length, with a LSC region of 79,992 bp, an SSC of 12,572 and two inverted repeats (IRa and IRb) each with a length of 21,388 bp. The average GC content was 38.27% (Table 1, Figure 1).

**Figure 2:**
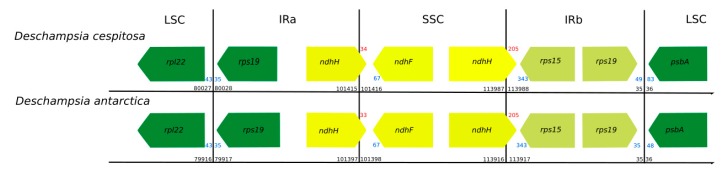
Comparison of the boundaries between genome regions in *Deschampsia cespitosa* and *D. antarctica* (black numbers: region boundaries; red numbers: gene crossing region boundary; blue numbers: distance from gene end to region boundary. All numbers in bp. Direction of arrow indicates direction of transcription).

The cp genome of *D. cespitosa* contains 115 genes, including 81 protein-coding genes, four rRNA genes and 30 tRNA genes (Table S1). Twelve genes are duplicated in the IR and there are three open reading frames (ORF 188, ORF 56 and ORF 42). Gene order and size are nearly identical to those of *D. antarctica*. Both copies of the *ndhH* gene cross the boundaries between the regions: 34 bp in the IRa-SSC and 205 bp in the SSC-IRb (Figure 2). Of the 30 tRNA genes, 21 are located in the LSC, with *trnT*-GGU duplicated-. Furthermore, one gene is found in the SSC and the remaining eight are in the IRs. The four rNA genes are located in the inverted repeats, with two in each IR. Some functional groups are located in the LSC region, i.e., the small subunit of ribosome (*rps* genes, except *rps15* in the SSC and *rps7* and *12* in the IR), the large subunit (*rpl* genes except *rpl32*), the RNA polymerase (*rpo* genes), photosystems I (*psa* genes except *psaC*) and II (*psb* genes) and the cytochrome complex (*pet* genes). The NADH (nicotinamide adenine dinucleotide) dehydrogenase (*ndh*) genes are located in the SSC region, except for *ndhB* that is transpliced between the two IRs.

### 2.3. Repetitive Sequences

**Figure 3:**
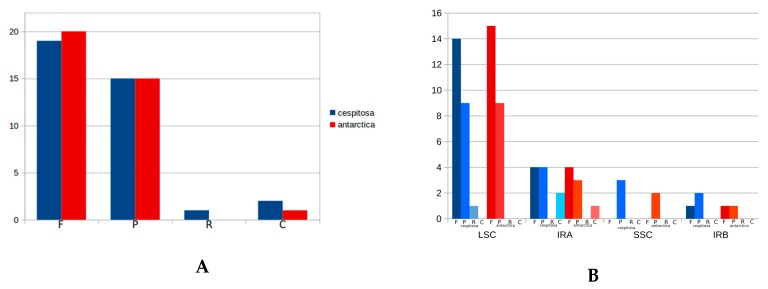
(**A**) Type of repeat sequences in plastid genomes of *Deschampsia cespitosa* and *D. antarctica* (F: forward; P: palindrome; R: reverse; C: complementary); (**B**) Location of repeat sequences in genomic regions of plastid genomes in *Deschampsia cespitosa* and *D. antarctica* (LSC: Long single- copy; SSC: Short single-copy; IRA: Inverted repeat A; IRB: Inverted repeat B).

The cp genome of *Deschampsia cespitosa* contains 37 repeats (19 forward, 15 palindrome, two complementary and one reverse) (Table S2). Repeats were mostly located in intergenic spacers and coding sequences; a single repeat was found in non-coding sequences. A comparison with repeat sequences in *D. antarctica* is shown in Figure 3A,B. The length of repeats varies between 19 and 224.

### 2.4. RNA Editing Sites

There are overall 41 RNA editing sites in 15 genes of the cp genome of *D. cespitosa* (Table S3). Most of the sites (18) were found in the *ndhB* gene, which is transpliced between the inverted repeats (nine sites each in IRa and IRb). Other genes with several editing sites include *rpoC2* (LSC, five sites), *matK*, *ndhA* and *rpoB* (LSC, four sites).

### 2.5. Phylogenomic Comparison

**Figure 4:**
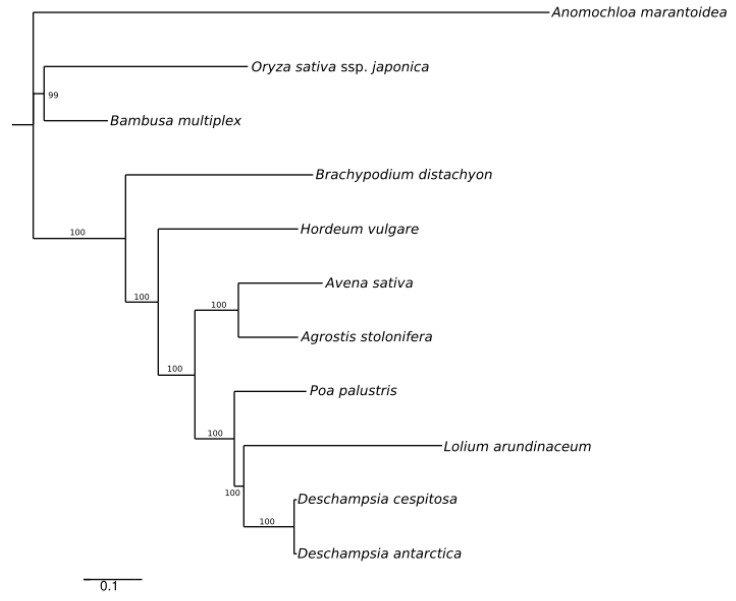
ML phylogenetic tree based on plastid genomes of *Deschampsia cespitosa* and selected grass species.

A maximum likelihood (ML) reconstruction with 11 selected whole cp genome sequences (Table S4) yielded a single tree with high support (Figure 4), depicting the low divergence between *Deschampsia cespitosa* and *D. antarctica*.

### 2.6. Genomic Comparison of Deschampsia cespitosa with D. antarctica and other Poaceae

**Figure 5:**
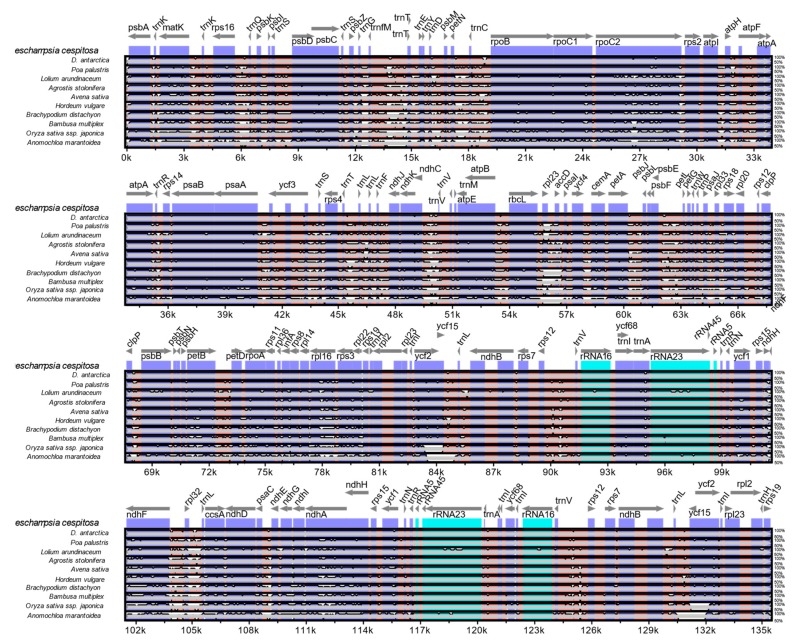
Complete cp genome comparison of eleven species of Poaceae.

**Figure 6:**
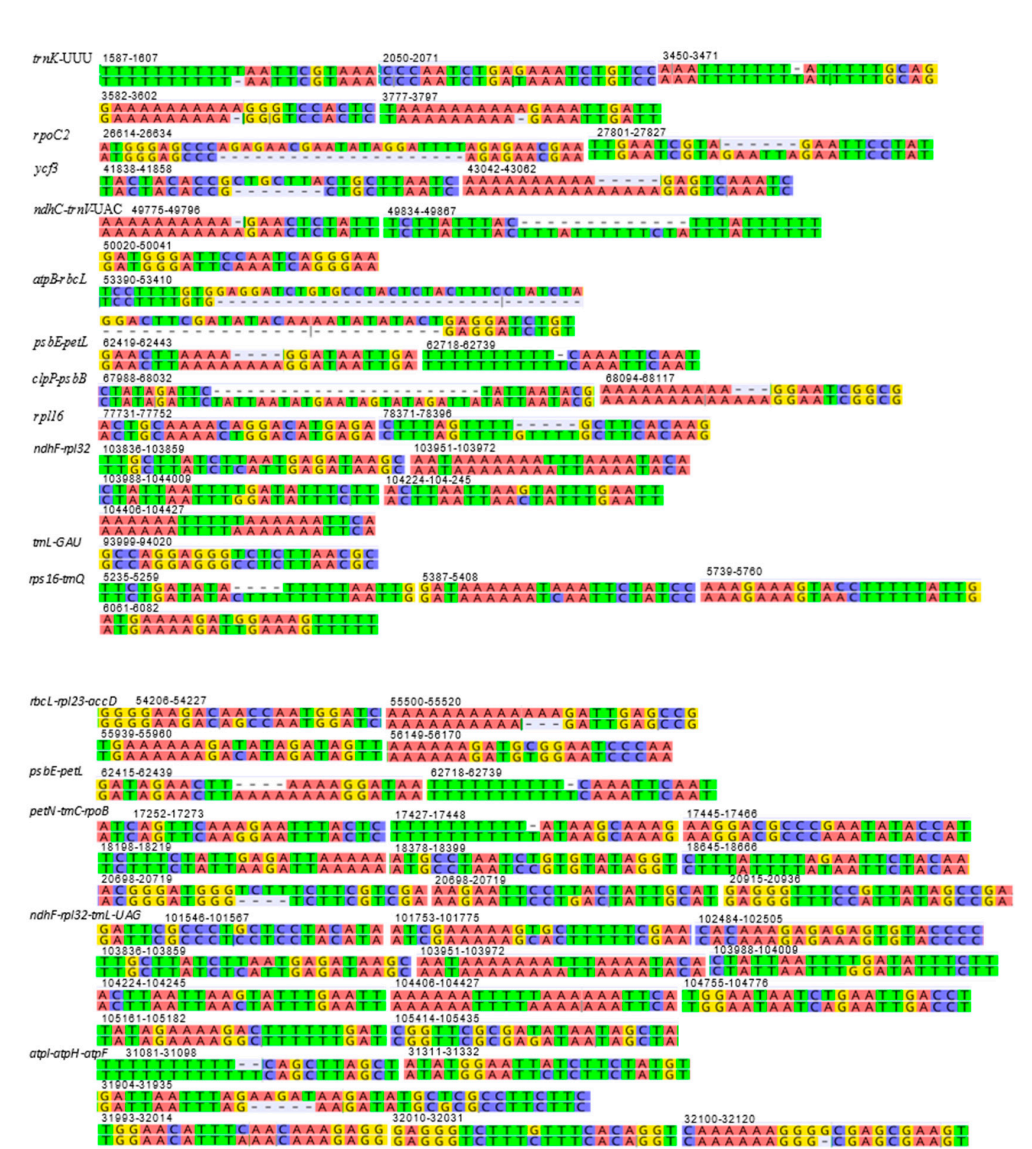
Structural differences (mutational differences, insertions/deletions and inversions) of plastid genome between *Deschampsia cespitosa* and *D. antarctica* of selected regions of interest with an additional 10 bp at both flanking regions. Numbers indicate the position in the *D. cespitosa* plastid genome.

The mVISTA alignment of 11 plastid (details in Table S4) sequences shows that conservation decreases progressively among the BEP clade species towards the early-diverging *Anomochloa maranthoidea* (Figure 5; Figure S1). The mVISTA alignment shows potential variable sites between *D. cespitosa* and the other Poaceae in *trnK*, *trnL*, *trnC rpoC2*, *rps14* and *rpl*16. Specifically focusing on differences in the plastid genome between *Deschampsia cespitosa* and *D. antarctica,* Figure 6 shows the structural differences (mutational differences, indels: insertions/deletions and inversions) of selected markers with both flanking regions in *trnK*-UUU, *rpoC2*, *ycf3*, *ndhC*-*trnV*-UAC, *atpB*-*rbcL*, *psbE*-*petL*, *clpP*-*psbB*, *rpl16*, *ndhF*-*rpl32*, *trnI*-GAU, *rps16*-*trnQ*, *rbcL*-*rpl23*-*accD*, *psbE*-*petL*, *petN*-*trnC*-*rpoB*, *ndhF*-*rpl32*-*trnL*-UAG and *atpl*-*atpH*-*atpF*. Most variations between *Deschampsia cespitosa* and *D. antarctica* are single mutations and small indels, which are more frequent in the intergenic spaces than in genes. Larger indels were found predominantly in intergenic spacers and to a lesser extent in coding regions, i.e., *rpoC2* and *ycf3*.

### 2.7. Microsatellites

There are 332 SSRs in the cp genome of *Deschampsia cespitosa* and 327 in *D. antarctica* (Table S5).

### 2.8. Codon Usage

Codon usage in protein-coding sequences Figure 6 showed a rather unimodal distribution, which is a common feature in grasses [24].

## 3. Discussion

**Figure 7:**
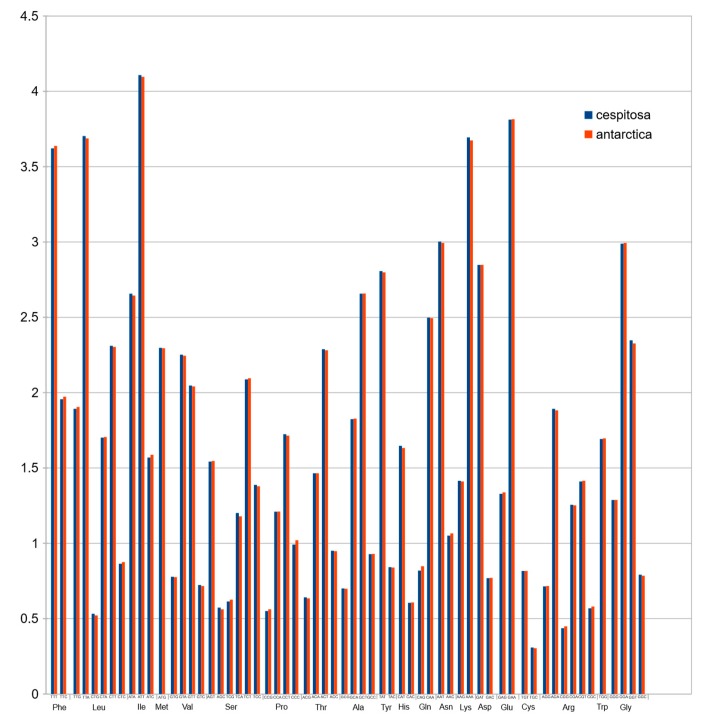
Normalized codon usage of *Deschampsia cespitosa* and *D. antarctica*.

Plastid genomes are uniparentally inherited [12], and the present study highlights its high conservation between two species of the genus *Deschampsia*. The comparison of genome size, gene order, repetitive sequences and codon usage between *D. cespitosa* and *D. antarctica* shows a high degree of similarity (Figure 7). Furthermore, the phylogenetic reconstruction shows little divergence between the two. A comparison per region of G-C content also shows essentially the same values (Table 1). The mVISTA alignment of the whole cp sequences (Figure 5; Figure S1) provides informative sites for phylogenetic and phylogeographic studies in the BEP clade. There are several mutations, indels and inversions that can be used for phylogeographic studies in the genus *Deschampsia.* Figure 6 shows structural differences in the plastid genome between *Deschampsia cespitosa* and *D. antarctica* of selected regions and their flanking regions. The main differences were detected in *trnK-UUU, rpoC2, ycf3, ndhC-trnV-UAC, atpB-rbcL, psbE-petL, clpP-psbB, rpl16, ndhF-rpl32* and *trnI-GAU.* Some genes, i.e., *rpoC2* and *ycf3*, have large indels differing between *Deschampsia cespitosa* and *D. antarctica*, which can be used in phylogenetic studies. Dispersed repeats of the forward, palindrome and reverse type are more common in the LSC region of both taxa, whereas only *D. antarctica* has a complementary repeat in the IRa region. The repeats are also more common in intergenic regions (Table S2). Codon usage in protein-coding sequences is rather similar between the two species and shows a bias to having an A in the third position [24]. The similarity of microsatellites between the two species (Table S3) allows for comparative mapping [25], especially in sympatric populations in Patagonia [23]. The major genomic region boundaries (Figure 2) are also highly similar and show few bp displacements between the two species.

## 4. Materials and Methods

### 4.1. Sample Material, DNA Extraction and Sequencing

The individual *Deschampsia cespitosa* used for this analysis was collected in a clearing of a spruce forest in the Karawanken mountain range, Carinthia, Austria (a voucher specimen is deposited in the herbarium WU). Leaves were dried in silica gel and DNA was extracted from 20 mg of dry tissue with the DNeasy Plant Mini Kit (Qiagen, Hilden, Germany), following the manufacturer’s protocol. The total DNA was visualized with agarose gel electrophoresis on a transilluminator Gel Doc 2000 (Biorad, Vienna, Austria) while its quality and quantity were assessed using a Nanodrop 2000 spectrophotometer (Thermo Fisher Scientific, Vienna, Austria) and a Qubit 2.0 (Thermo Fisher Scientific). Whole cellular DNA (nuclear, mitochondrial and plastid DNA) was sheared with a Bioruptor^®^ Pico sonication device (Diagenode, Liege, Belgium) using seven cycles of 15 s on and 90 s off at 4 °C in order to obtain fragments with an average size of 350 to 400 bp. Fragment size was checked afterwards with an Agilent 2100 Bioanalyzer (Agilent Technologies, Santa Clara, CA, USA). The library was prepared with a TruSeq DNA PCR Free Library Kit (Illumina, San Diego, CA, USA), following the manufacturer’s protocol and barcodes provided with the kit. The library was sequenced in a 1/24 lane of an Illumina HiSeq 3000 at VBCF Vienna (https://www.vbcf.ac.at/facilities/next-generation-sequencing/).

### 4.2. Genome Assembly and Annotation

The BAM file with total genomic DNA sequences was downloaded from the facility and sorted with Bedtools [26] before fastq files were produced with Samtools [27]. Fastq files were processed with Fast-Plast [28] to assemble the cp genome. The Fast-Plast pipeline includes Trimmomatic [29], which performs an initial read cleaning; Bowtie2 [30], which reduces reads to only plastid-like reads; and SPAdes [31], which performs an initial assembly. The obtained genome was then annotated online using DOGMA [32] with default parameters, which used BLASTX and BLASTN searches to identify all genes by comparing them with a custom database of published cp genomes. The phylogenetically closest complete cp genome of *D. antarctica* was utilized to confirm the positions of start and stop codons and boundaries of exons or introns by alignment in the software Geneious 11.1.5 [33]. The annotated genome was finally uploaded in Genome VX [34] to produce the circular map (Figure 1). The complete cp genome was deposited in GenBank (GenBank accession MK262782).

### 4.3. Repeats Structure

Simple repetitive short sequences with repeated motifs of 1–10 bp are a common feature of plant genomes [35] that can be used in population genetics or to tag genes of interest [36]. Repeats (forward, palindrome, reverse and complement sequences) in the cp genome (Table S2) were detected with the REPuter server [37].

### 4.4. RNA Editing Sites

RNA editing sites are codon positions in mitochondrial and cp sequences, at which potential changes of C-to-U could result in changes in the encoded amino acid [38]. The editing sites in the cp genome of *Deschampsia cespitosa* (Table S3) were identified with the PREP (Predictive RNA Editor for Plants) suite, which is a set of web servers devoted to predicting such sites in plant organellar genes [38].

### 4.5. Phylogenetic Analysis

To verify the phylogenomic relationships of the newly obtained plastome of *Deschampsia cespitosa* in relation to other Poaceae, we selected early-diverging Poaceae and representatives of the BEP clade, especially members of the tribe Poeae ([7,39]; *Agrostis stolonifera* [12], *Anomochloa maranthoidea* [40], *Avena sativa* [41], *Bambusa multiplex* [42], *Brachypodium distachyon* [43], *D. antarctica* [6], *Hordeum vulgare* ssp. *vulgare* [12], *Lolium arundinaceum* [44], *Oryza sativa* ssp. *japonica* [11] and *Poa palustris* [41]) (Table S4). The data matrix was aligned with MAFFT [45] using standard settings. The alignment obtained with MAFFT was visually controlled with BioEdit [46]. The aligned matrix was uploaded in the IQ-TREE server [47] before a ML tree search with bootstrap support was run. The resulting tree (Figure 4) was edited with Inkscape [48].

### 4.6. Comparative Analyses

The comparative genomic analysis of *Deschampsia cespitosa*, early-diverging Poaceae and members of the BEP clade (Table S1) was conducted with mVISTA [49], which is a server for comparative analysis of genomic-level sequence. We used default parameters with the new sequence of *D. cespitosa* set as the reference (Figure 5; Figure S1). Comparisons between *Deschampsia cespitosa* and *D. antarctica* were made with Geneious [33].

### 4.7. Microsatellite Search

Microsatellites are ubiquitous components of all genomes [50] and are extremely variable [51]. We used Imperfect Microsatellite Extractor [52], which is an online server tool for finding microsatellites, Simple Sequence Repeats (SSRs) or Short Tandem Repeats (STRs) from genomic sequences, to detect microsatellites (Table S3). We followed a previous study [3] to create a similar comparison between two related species, setting minimum thresholds for search at seven for mononucleotide repeats, four for dinucleotide repeats and three for tri-, tetra-, penta- and hexanucleotide repeats.

### 4.8. Codon Usage

The codon usage percentage of protein coding sequences was estimated with Sequence Manipulation Suite, an online collection of Java Scripts for analyzing short DNA and protein sequences, using default settings. [53].

## 5. Conclusions

The results suggest a high similarity of the plastid genome of *Deschampsia cespitosa* and *D. antarctica*. The major genomic region boundaries (Figure 2) are highly similar and the few differences suggest a relatively stable cp genomic structure in the genus; however, the inclusion of further cp genomes of other species of the genus and *D. cespitosa* samples from other geographical regions is necessary to verify this. The similarity of the obtained cp genome may also highlight the possibility of using *D. cespitosa* for research in any of the fields mentioned in the introduction, which are currently restricted to *D. antarctica*. Available evidence [39] pointing to an origin of the genus in Eurasia and the worldwide distribution of *D. cespitosa* may reveal *D. antarctica* to be a cold-adapted form derived from the former once we can include more members of the genus in the analysis.

## Figures and Tables

**Table 1:** Plastid genomes of *Deschampsia cespitosa* and *D. antarctica* (from [5]).

Feature	*Deschampsia cespitosa*	*Deschampsia antarctica*
Size (bp)	135,340	135,362
LSC (bp)	79,992	79,881
SSC (bp)	12,572	12,519
IR (bp)	21,388	21,481
GC content total (%)	38.28	38.30
GC content LSC (%)	36.23	36.30
GC content SSC (%)	32.42	32.40
GC content IR (%)	43.81	43.85

## Supplementary Materials

The supplementary materials are available online.
