# Supplementary material for: The Plastid Genome of Deschampsia cespitosa (Poaceae)
Source: Molecules. 2019 Jan 9;24(2):216. doi: 10.3390/molecules24020216 (PMC6359331; doi:10.3390/molecules24020216)
Supplement: Supplementary file 1 [file molecules-24-00216-s001.zip › molecules-401694-supplementary material/Suppl-Table 1.pdf]

Table S1. Comparison of gene functional groups of *Deschampsia antarctica* and *D. cespitosa.*

Gene group	*Deschampsia cespitosa*	*Deschampsia antarctica*
Small subunit of ribosome	*rps*2, *rps*3, *rps*4, *rps*7[3], *rps*8, *rps*11, *rps*12[2,3], *rps*14, *rps*15[3], *rps16*[2], *rps18*, *rps19*[3]	=
Large subunit of ribosome	*Rpl*2[2,3], *rpl*14, *rpl*16[2], *rpl*20, *rpl*22, *rpl*23[3], *rpl*32, *rpl*33, *rpl*36	=
RNA polymerase subunits	*rpo*A, *rpo*B, *rpo*C1, *rpo*C2	=
NADH dehydrogenase	*ndh*A[2], *ndh*B[2,3], *ndh*C[3], *ndh*D, *ndh*E, *ndh*F, *ndh*G, *ndh*H[3], *ndh*I, *ndh*J, *ndh*K	=
Photosystem I	*psa*A, *psa*B, *psa*C, *psa*I, *psa*J, *ycf*3[1], *ycf*4	=
Photosystem II	*psb*A, *psb*B, *psb*C, *psb*D, *psb*E, *psb*F, *psb*H, *psb*I, *psb*J, *psb*K, *psb*L, *psb*M, *psb*N, *psb*T, *psb*Z	=
Cytochrome b/f complex	*pet*A, *pet*B[2], *pet*D[2], *pet*G, *pet*L, *pet*N	=
ATP synthase	*atp*A, *atp*B, *atp*E, *atp*F[2], *atp*H, *atp*I	=
Large subunit of rubisco	*rbc*L	=
Maturase	*mat*K	=
Protease	*clp*P	=
Envelope membrane protein	*cem*A	=
Subunit of acetyl-Coa-carboxylase	*acc*D	=
C-type cytochrome	*ccs*A	=
TIC complex	*Ycf*1[3,4]	=
Hypothetical chloroplast reading frame	*ycf2*[3]	=
Translation initiation factor	*inf*A	=
ORFs	*ycf*15[3], *ycf*68[3]	=
Ribosomal RNA genes	*rRNA*4.5[3], *rRNA*5[3], *rRNA*16[3], *rRNA*23[3]	=
Transfer RNA genes	*trnA(UGC)*[2], *trnC(GCA), trnD(GUC), trnE(UUC), trnF(GAA), trnG(UUC), trnH(GUG), trnI(CAU), trnI(GAU)*[2], *trnK(UUU)*[2] *trnL(UAA)*[2], *trnL(UAG) trnL(CAA), trnfM(CAU) trnM(CAU), trnN(GUU) trnP(UUG), trnQ(UUG) trnR(ACG), trnR(UCU) trnS(GCU), trnS(GGA) trnS(UGA), trnT(GGU) trnT(UGU), trnV(GAC) trnV(UAC)*[2], *trnW(CCA) trnY(GUA)*	=

[1] Gene with two introns.
[2] Gene with one intron.
[3] Two copies in IR.
[4] Pseudogene.
