# Supplementary material for: The Plastid Genome of Deschampsia cespitosa (Poaceae)
Source: Molecules. 2019 Jan 9;24(2):216. doi: 10.3390/molecules24020216 (PMC6359331; doi:10.3390/molecules24020216)
Supplement: Supplementary file 1 [file molecules-24-00216-s001.zip › molecules-401694-supplementary material/Suppl-Table 2.pdf]

Table S2. Size, type and positions of long repetitive sequences in the plastid genome of *Deschampsia cespitosa*. Columns from left to right: repeat size (bp), type of repeat (F, forward; P, palindrome; R, reverse; C, complement); start position and position of the repetition; location.

Size	Type	Start, repeat	Location	Size	Type	Start, repeat	Location
224	F	55663,133028	*rpl*23 - intergenic	224	P	55663,82150	intergenic - intergenic
39	F	55888,133253	*rpl*23 - intergenic	39	P	55888,82110	*rpl*23 - intergenic
30	F	86640,128732	no coding - intergenic	36	P	1,80026	intergenic - intergenic
29	F	100621,114783	no coding - intergenic	29	P	114752,114783	intergenic - intergenic
29	F	100590,114752	intergenic - intergenic	29	P	100590,100621	intergenic - intergenic
29	F	26538,26586	intergenic - intergenic	29	P	7624,43973	intergenic - intergenic
26	F	75827,75845	*inf*A- *inf*A	24	P	104964,104994	intergenic - intergenic
26	F	65264,65306	*rps*18 - *rps*18	24	P	14371,126406	intergenic - intergenic
26	F	26602,26656	intergenic - intergenic	23	P	103824,103848	intergenic - intergenic
24	F	14371,88972	intergenic - intergenic	21	P	90441,124959	intergenic - intergenic
23	F	62596,62610	intergenic - intergenic	21	P	90422,124940	intergenic - intergenic
21	F	124940,124959	intergenic - intergenic	21	P	14811,45184	intergenic - trnT(UGU)
21	F	90422,90441	intergenic - intergenic	21	P	11268,43976	*trn*S(UGA)- *trn*S(GGA)
21	F	26526,26649	intergenic - intergenic	19	P	46995,121061	*trn*F(GAA) - *trn*A(UGC)
21	F	7629,11268	intergenic - intergenic	19	P	34,59	intergenic - intergenic
20	F	37559,39783	intergenic - intergenic	19	R	43145,47062	*ycf*3 - *trn*F(GAA)
20	F	24604,24625	intergenic - intergenic	19	C	100841,114542	*rps*15 - intergenic
19	F	46995,94322	*trn*F(GAA) - *trn*A(UGC)	19	C	84188,131195	*ycf*15 - *ycf*15
19	F	6433,18019	*trn*Q(UUG) - *trn*C(GCA)				
