# Supplementary material for: The Plastid Genome of Deschampsia cespitosa (Poaceae)
Source: Molecules. 2019 Jan 9;24(2):216. doi: 10.3390/molecules24020216 (PMC6359331; doi:10.3390/molecules24020216)
Supplement: Supplementary file 1 [file molecules-24-00216-s001.zip › molecules-401694-supplementary material/Suppl-Table 3.pdf]

Table S3. RNA editing sites in the plastid genome of *Deschampsia cespitosa*.

Gene	Start	End	Cp genome region	RNA edit site	Nt Pos	AA pos	Effect	Score
psbA	123	1184	LSC	1	65	22	ACT (T) => ATT (I)	1.00
			LSC	2	169	57	CA (P) => TCA (S)	1.00
			LSC	3	250	84	CCT (P) => TCT (S)	1.00
			LSC	4	464	155	ACT (T) => ATT (I)	0.80
			LSC	5	754	252	CAT (H) => TAT (Y)	0.80
			LSC	6	857	286	ACT (T) => ATT (I)	1.00
matK	1724	3965	LSC	7	350	117	ACC (T) => ATC (I)	0.86
			LSC	8	355	119	CTC (L) => TTC (F)	0.86
			LSC	9	373	125	CCT (P) => TCT (S)	1.00
			LSC	10	1282	428	CAT (H) => TAT (Y)	1.00
atpB	51642	53138	LSC	11	1487	496	TCG (S) => TTG (L)	1.00
ndhA ex1	110910	111449	SSC	12	14	5	TCA (S) => TTA (L)	1.00
			SSC	13	13521	174	TCC (S) => TTC (F)	1.00
ndhA ex2	112462	113010	SSC	14	50	17	TCG (S) => TTG (L)	1.00
			SSC	15	473	158	TCA (S) => TTA (L)	1.00
ndhB ex1	127429	128205	IrB	16	53	18	TCA (S) => TTA (L)	1.00
			IrB	17	59	20	TCA (S) => TTA (L)	1.00
			IrB	18	704	235	CCA (P) => CTA (L)	1.00
ndhB ex2	128918	129673	IrB	19	149	50	TCA (S) => TTA (L)	1.00
			IrB	20	467	156	CCA (P) => CTA (L)	1.00
			IrB	21	586	196	CAT (H) => TAT (Y)	1.00
			IrB	22	611	204	TCA (S) => TTA (L)	0.80
			IrB	23	704	235	TCC (S) => TTC (F)	1.00

Table S3. RNA editing sites in the plastid genome of *Deschampsia cespitosa* (continued).

			IrB	24	737	246	CCA (P) => CTA (L)	1.00
ndhD	106732	108234	SSC	25	878	293	TCA (S) => TTA (L)	1.00
ndhF	101483	103702	SSC	26	62	21	TCA (S) => TTA (L)	1.00
			SSC	27	1697	566	GCA (A) => GTA (V)	1.00
petB	70854	72260	LSC	28	620	207	CCA (P) => CTA (L)	1.00
rpl2 ex 2	81700	82092	IrA	29	2	1	ACG (T) => ATG (M)	1.00
rpl20	65895	66254	LSC	30	308	103	TCA (S) => TTA (L)	0.86
rpoA	73878	74903	LSC	31	235	79	CTC (L) => TTC (F)	0.80
rpoB	19164	22394	LSC	32	467	156	TCG (S) => TTG (L)	0.86
			LSC	33	545	182	TCA (S) => TTA (L)	1.00
			LSC	34	560	187	TCG (S) => TTG (L)	1.00
			LSC	35	617	206	CCG (P) => CTG (L)	0.86
rpoC2	24666	29057	LSC	36	1297	433	CCC (P) => TCC (S)	0.86
			LSC	37	296	99	GCA (A) => GTA (V)	0.86
			LSC	38	1061	354	CCG (P) => CTG (L)	1.00
			LSC	39	815	272	TCA (S) => TTA (L)	0.86
rps8	76251	76661	LSC	40	182	61	TCA (S) => TTA (L)	0.86
ycf3	41312	41470	LSC	41	44	15	TCC (S) => TTC (F)	1.00
