# Supplementary material for: The Plastid Genome of Deschampsia cespitosa (Poaceae)
Source: Molecules. 2019 Jan 9;24(2):216. doi: 10.3390/molecules24020216 (PMC6359331; doi:10.3390/molecules24020216)
Supplement: Supplementary file 1 [file molecules-24-00216-s001.zip › molecules-401694-supplementary material/Suppl-Table 4.pdf]

Table S4. Taxa included with GenBank accession and reference.

Taxon	GenBank	Reference
Agrostis stolonifera	NC008591	Saski et al., 2007 [12]
Anomochloa marantoidea	NC014062	Morris et al., 2010 [40]
Avena sativa	KM974733	Saarela et al., 2015 [41]
Bambusa multiplex	NC024668	Gao et al., 2016 [42]
Brachypodium distachyon	KU170609	Sancho et al., 2017 [43]
Deschampsia antarctica	KF887484	Lee et al., 2014 [6]
Deschampsia cespitosa	MK262782	(this study)
Hordeum vulgare ssp. *vulgare*	NC008590	Saski et al., 2007 [12]
Lolium arundinaceum	FJ466687	Cahoon et al., 2010 [44]
Oryza sativa japonica	AY522330	Tang et al., 2004 [11]
Poa palustris	KM974749	Saarela et al., 2015 [41]
