# Supplementary material for: The Plastid Genome of Deschampsia cespitosa (Poaceae)
Source: Molecules. 2019 Jan 9;24(2):216. doi: 10.3390/molecules24020216 (PMC6359331; doi:10.3390/molecules24020216)
Supplement: Supplementary file 1 [file molecules-24-00216-s001.zip › molecules-401694-supplementary material/Suppl-Table 5.pdf]

Table S5. Microsatellites in *Deschampsia cespitosa* and *D. antarctica*.

Deschampsia cespitosa														
Motif	***Number of repeats***													***Total***
	18	16	14	13	12	11	10	9	8	7	5	4	3	
A	1	1	1	1	1	1	3	21	30	67				127
T			1			5	8	12	29	62				117
C								1	3	6				10
G									2	6				8
AG/AT										1	1	7		9
CT												3		3
GA												3		3
TA/TC											3	4		7
AAC/AAG/AAT/AGA/AGT/ATA/ATT													16	16
CAA/CCT/CTT													3	3
GAA/GAT/GCA/GTT												1	5	6
TAA/TAT/TCC/TCT/TGC/TTA/TTC/TTG													15	15
AAAC/AGAA/ATAC													3	3
CAAA													1	1
TAAT													1	1
TTCA													1	1
TCCT													1	1
ATCCT													1	1
	1	1	2	1	1	6	11	34	64	142	4	18	47	332

Deschampsia antarctica												
Motif	***Number of repeats***											***Total***
	14	13	12	11	10	9	8	7	5	4	3	
A	1	3	1	2	7	16	27	66				123
T			2	3	6	16	26	62				115
C						1	3	6				10
G							2	6				8
AG/AT								1	1	8		10
CT										3		3
GA										3		3
TA/TC									3	4		7
AAC/AAG/AAT/AGA/AGT/ATA/ATT											16	16
CAA/CTT											2	2
GAT/GAA/GCA/GTT										1	5	6
TAA/TAT/TCC/TCT/TGC/TTA/TTC/TTG										1	14	15
AAAC/AGAA/ATAC											3	3
CAAA											1	1
TAAT											1	1
TTCA / TCCT											2	2
AAAAAG											1	1
TTTTCT											1	1
	1	3	3	5	13	33	58	141	4	20	46	327
