# Supplementary figures and images for: The Plastid Genome of Deschampsia cespitosa (Poaceae)
Source: Molecules. 2019 Jan 9;24(2):216. doi: 10.3390/molecules24020216 (PMC6359331; doi:10.3390/molecules24020216)

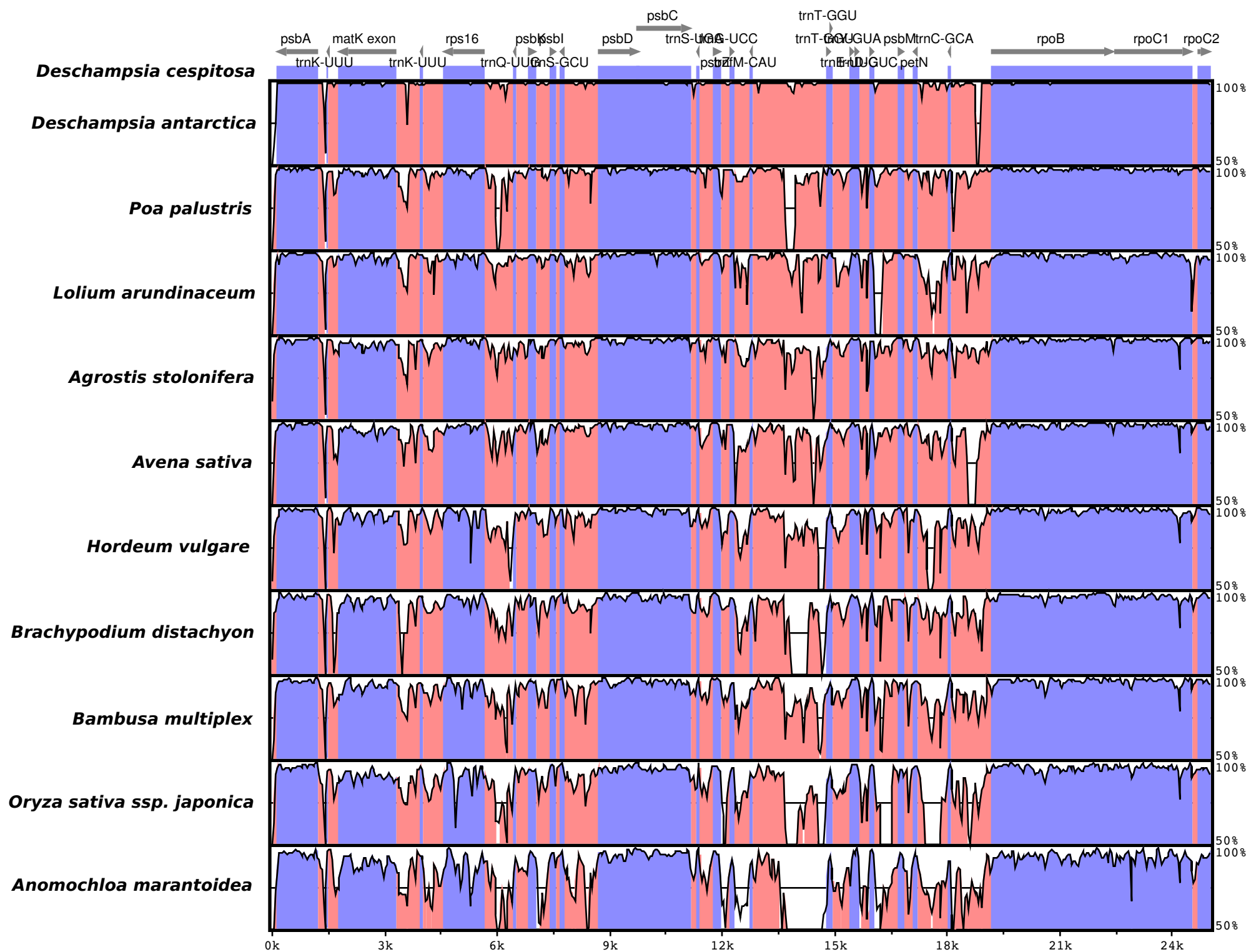

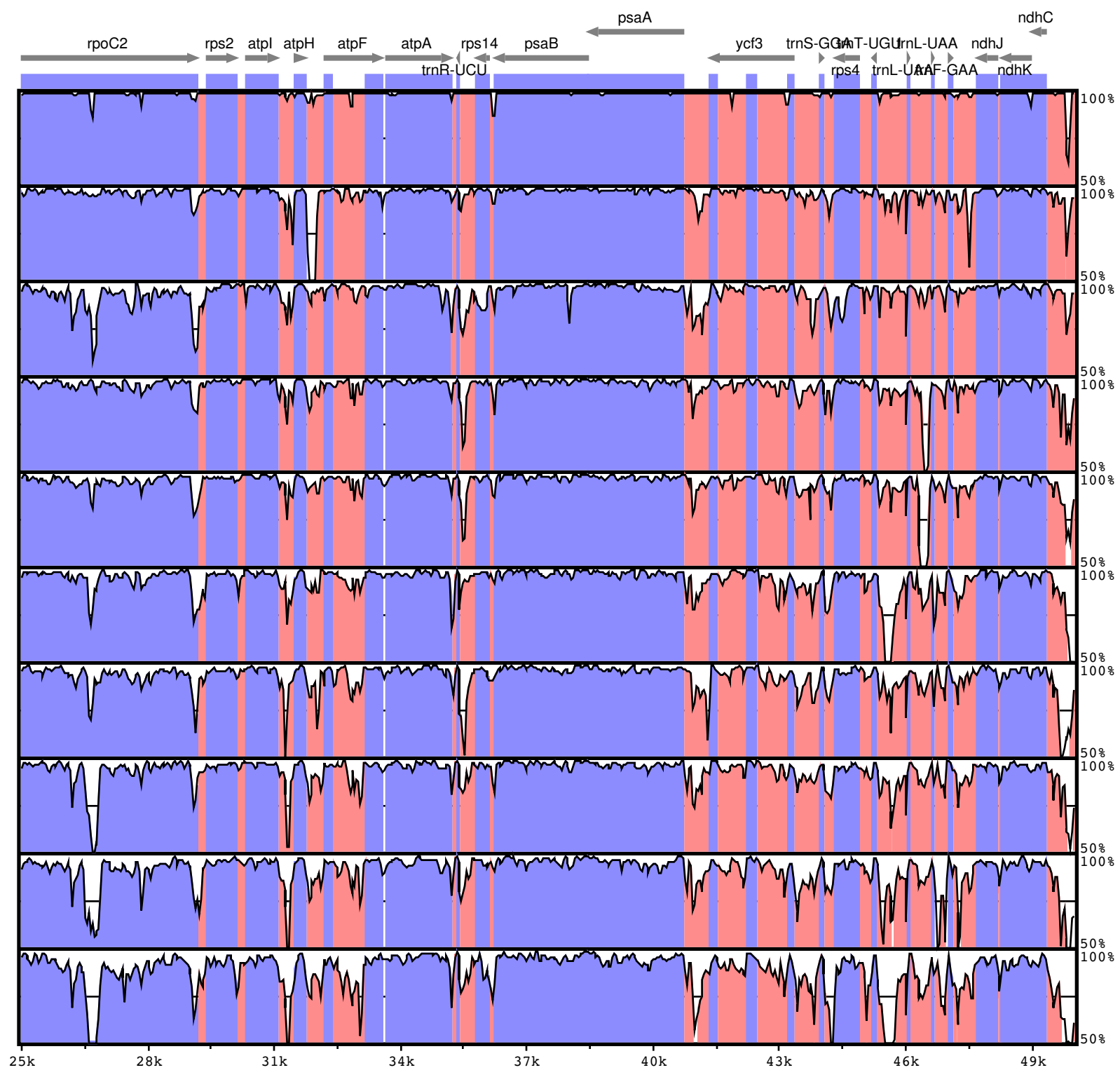

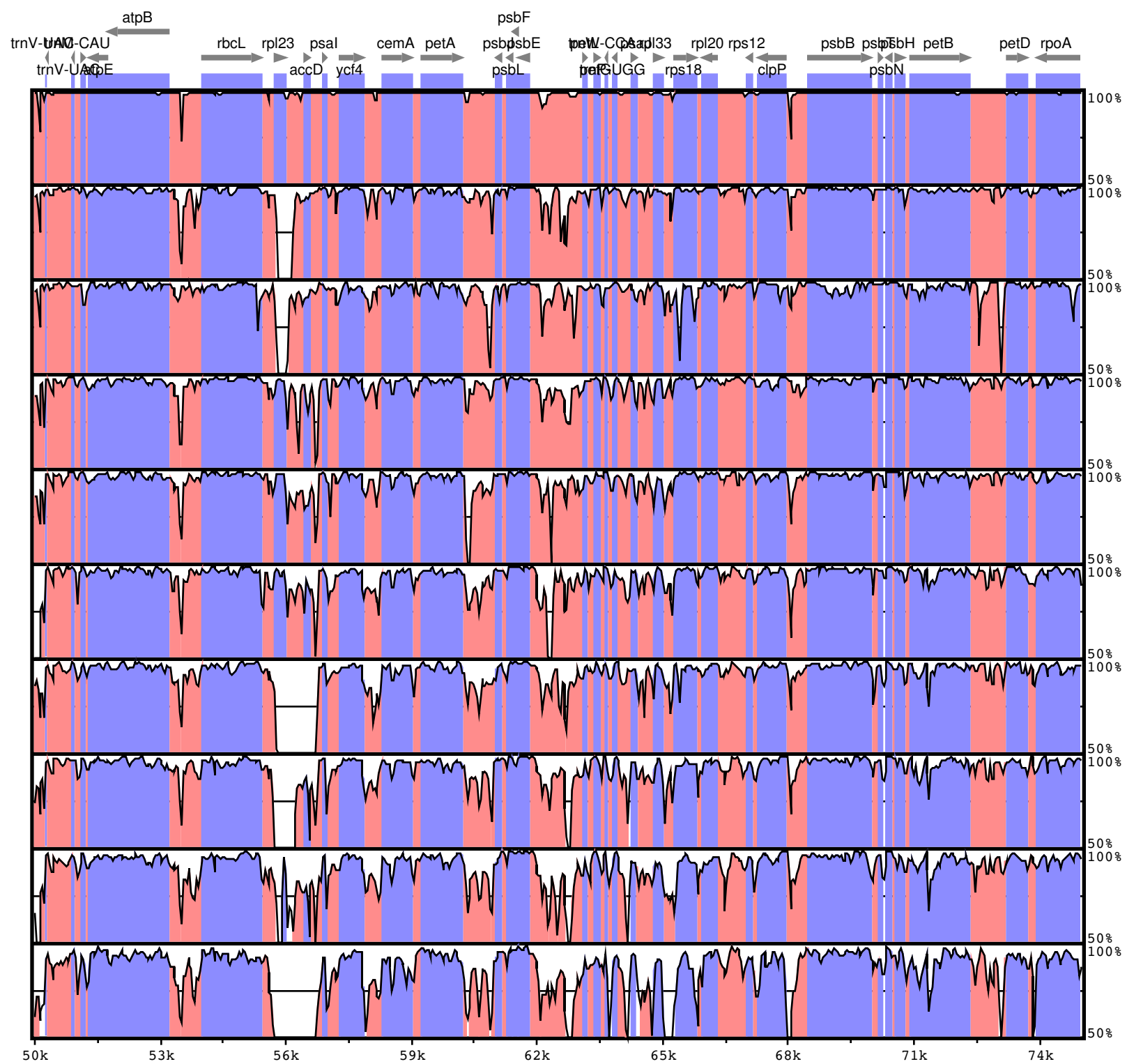

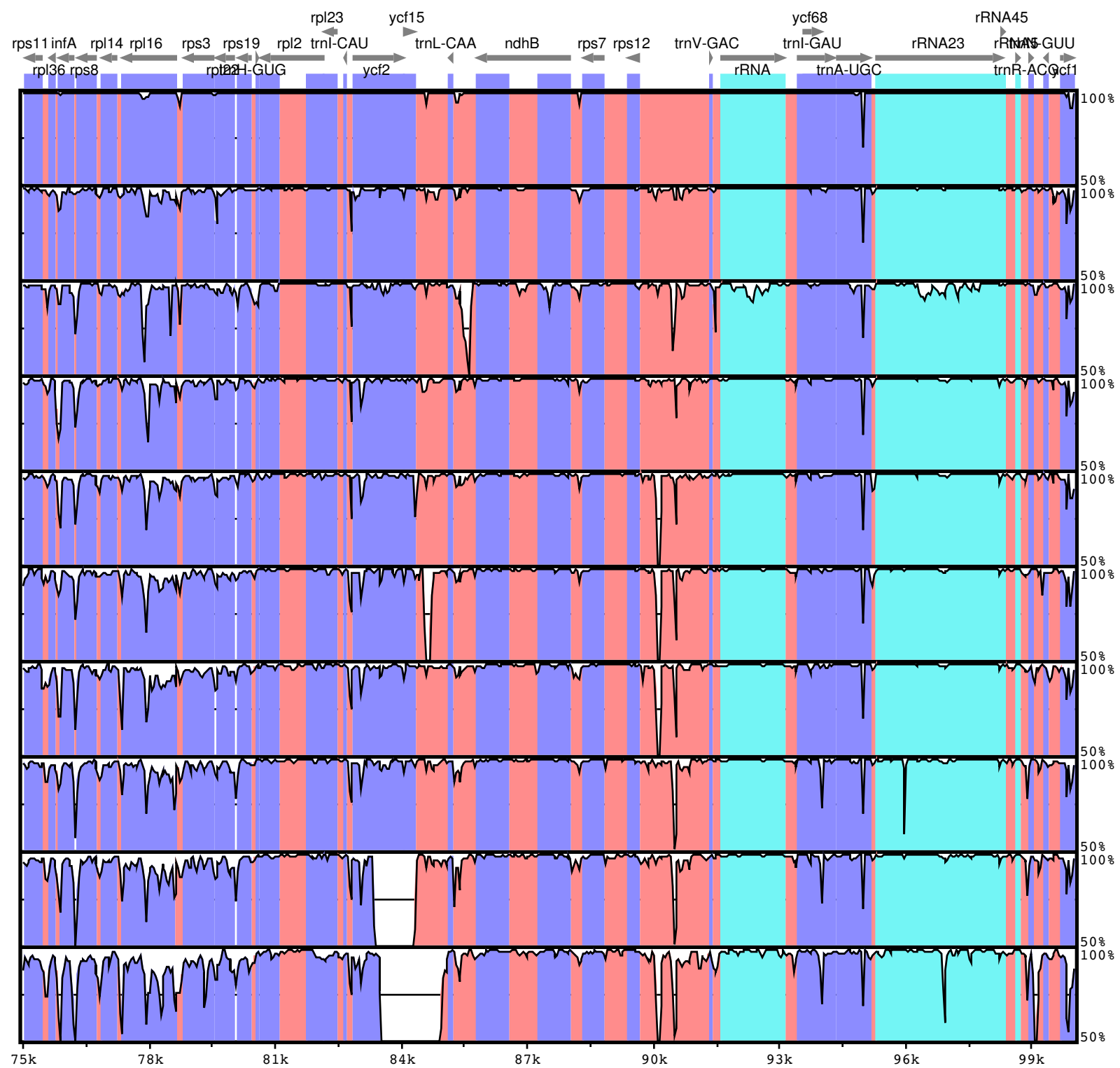

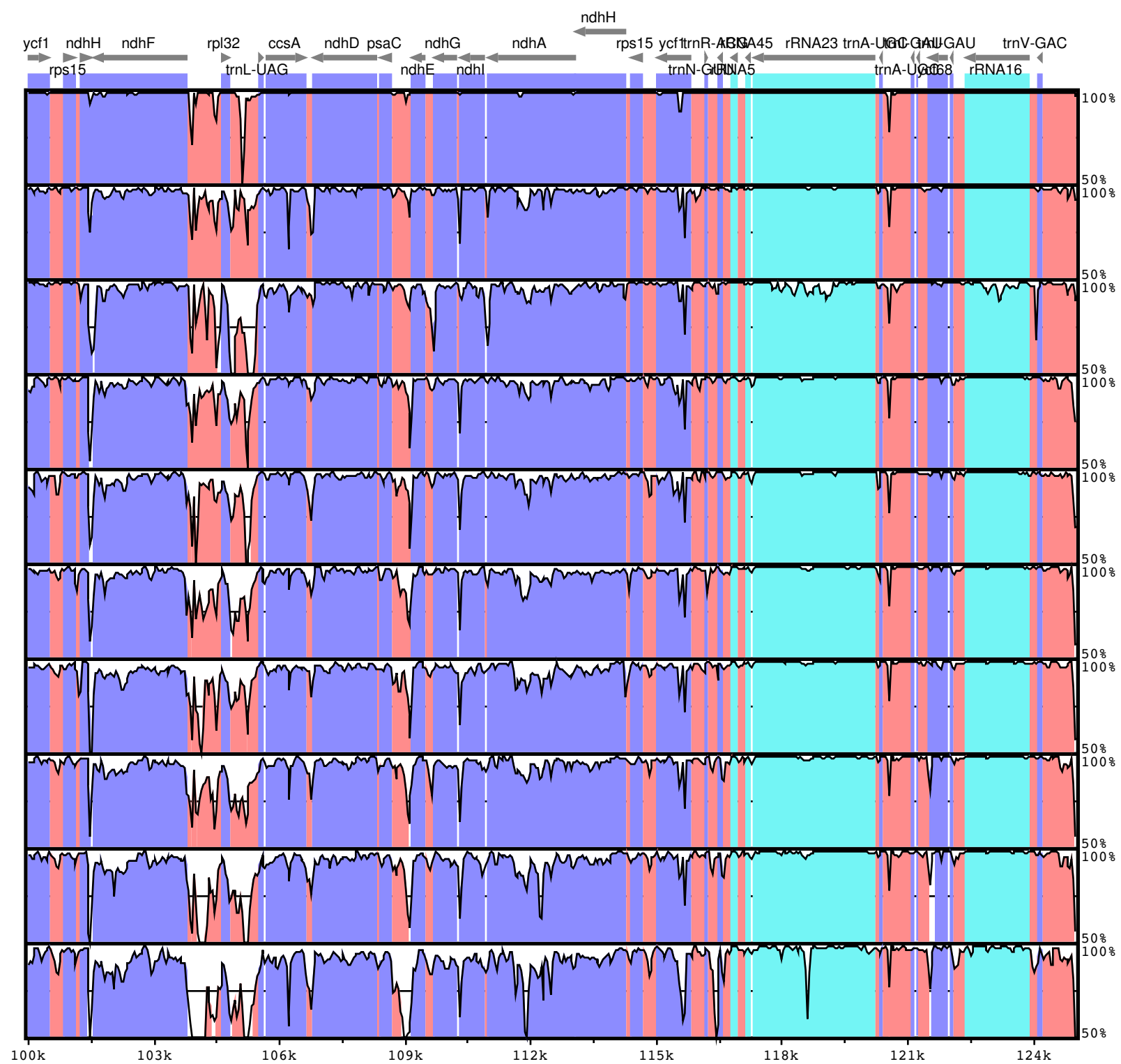

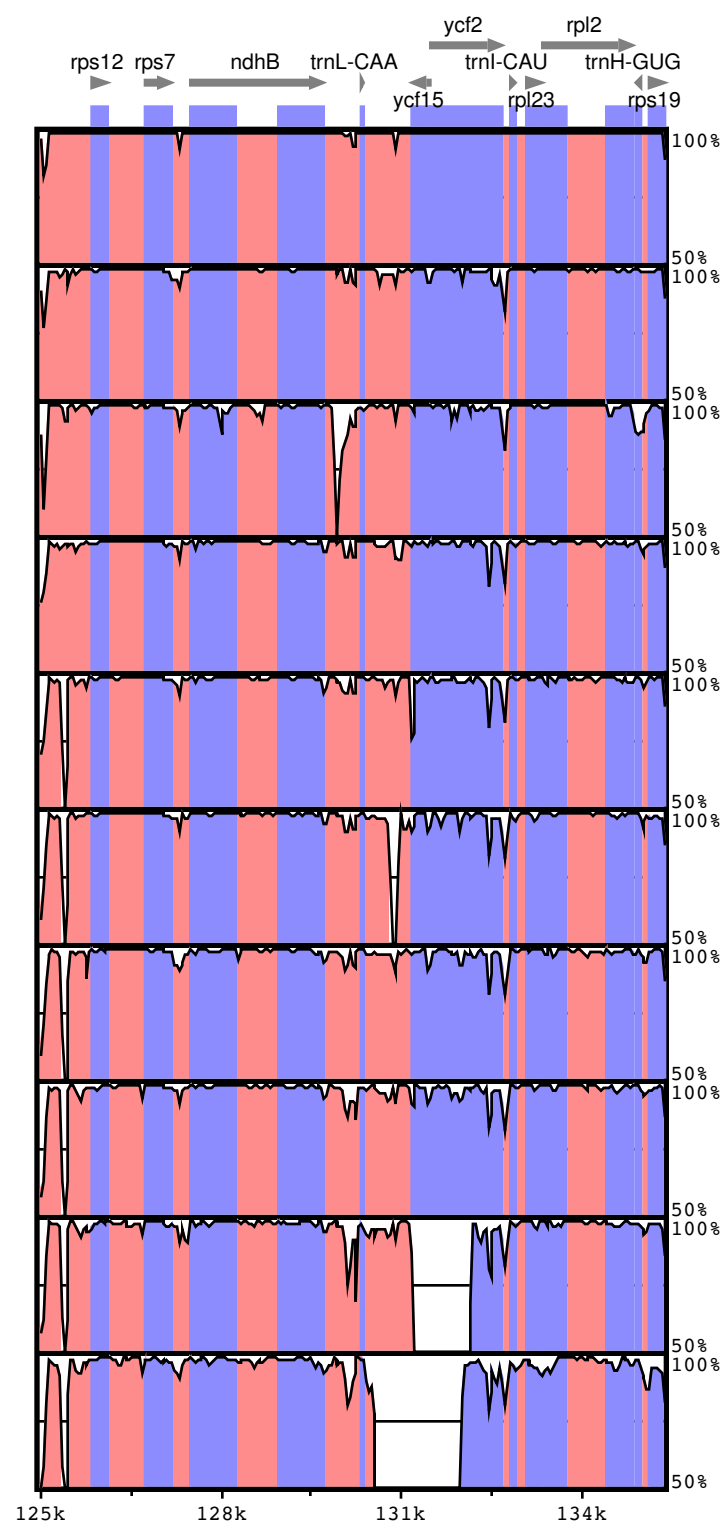

Supplement: Supplementary file 1 [file molecules-24-00216-s001.zip › molecules-401694-supplementary material/Suppl-Figure 1.pdf]
